# *Hamamelis virginiana* L. Leaf Extracts Inhibit the Growth of Antibiotic-Resistant Gram-Positive and Gram-Negative Bacteria

**DOI:** 10.3390/antibiotics12071195

**Published:** 2023-07-17

**Authors:** Matthew J. Cheesman, Sean R. Alcorn, Alan White, Ian E. Cock

**Affiliations:** 1 School of Pharmacy and Medical Sciences, Gold Coast Campus, Griffith University, Gold Coast 4222, Australia; m.cheesman@griffith.edu.au (M.J.C.); s.alcorn@griffith.edu.au (S.R.A.); 2 School of Environment and Science, Nathan Campus, Griffith University, Brisbane 4111, Australia; alan.white@griffith.edu.au

**Keywords:** witch hazel, plant extracts, antibiotic resistance, ESBL, MRSA

## Abstract

Virginian witch hazel (WH; *Hamamelis virginiana* L.; family: Hamamelidaceae) is a North American plant that is used traditionally to treat a variety of ailments, including bacterial infections. Solvents of varying polarity (water, methanol, ethyl acetate, hexane and chloroform) were used to prepare extracts from this plant. Resuspensions of each extract in an aqueous solution were tested for growth-inhibitory activity against a panel of bacteria (including three antibiotic-resistant strains) using agar disc diffusion and broth microdilution assays. The ethyl acetate, hexane and chloroform extracts were completely ineffective. However, the water and methanolic extracts were good inhibitors of *E. coli*, ESBL *E. coli*, *S. aureus*, MRSA, *K. pneumoniae* and ESBL *K. pneumoniae* growth, with the methanolic extract generally displaying substantially greater potency than the other extracts. Combining the active extracts with selected conventional antibiotics potentiated the bacterial growth inhibition of some combinations, whilst other combinations remained non-interactive. No synergistic or antagonistic interactions were observed for any WH extracts/antibiotic combinations. Gas chromatography–mass spectrometry analysis of the extracts identified three molecules of interest that may contribute to the activities observed, including phthalane and two 1,3-dioxolane compounds. Putative modes of action of the active WH extracts and these molecules of interest are discussed herein.

## 1. Introduction

The development of antibiotic-resistant bacterial pathogens is a serious threat to global human health. The World Health Organisation (WHO) estimates that by 2050, significantly more deaths will arise from antimicrobial resistance (AMR) annually than those caused by cancer [1]. Indeed, despite the ongoing SARS-CoV-2 pandemic, the WHO confirmed that AMR remained the leading worldwide public health threat facing humanity [2]. AMR does not discriminate on the basis of geographical location or income levels, and untreatable common infections are arising at increasingly alarming levels, resulting in elevations in patient morbidity and mortality [3,4].

It has been predicted that neonates and very young children will be especially susceptible to multidrug-resistant (MDR) pathogens. Indeed, infectious diseases already account for up to 40% of all child deaths, particularly in low- and middle-income countries [5]. For example, antibiotic-resistant neonatal infections account for approximately 60,000 newborn babies in India alone [6]. The most dangerous and lethal pathogens include the Gram-positive bacteria *Staphylococcus aureus*, as well as Gram-negative species, including *Escherichia coli* and *Klebsiella* spp. Cases of pneumonia arising from *S. aureus* infections in children are rising substantially, and the emergence of methicillin-resistant *S. aureus* (MRSA) is exacerbating the problem by increasing the rate of hospitalisations and deaths [7,8]. *Klebsiella pneumoniae* causes numerous types of respiratory, urinary tract and bloodstream infections [9], while *E. coli* is a prominent cause of diarrhoea in children [10]. Clinical isolates expressing extended-spectrum β-lactamases (ESBLs) have been identified in both of these organisms [11,12]. Notably, ESBL *K. pneumoniae* and *E. coli* strains display particularly high levels of resistance [13,14] and treatment options for infections caused by these organisms are limited [15].

The rapid emergence of AMR has led to a greatly reduced effectiveness of many classes of antibiotics. This has prompted investigations into new treatment modalities to treat infections caused by both antibiotic-susceptible and MDR bacterial species. Traditional plant-based medicines have gained significant interest in recent decades as targets for new drug development due to the growing evidence of their antimicrobial properties and their ability to enhance the effectiveness of conventional antibiotics [16]. Previous work from our group has reported that Virginian witch-hazel (WH) possesses inhibitory properties against Staphylococcal and Streptococcal strains [17], as well as against *Pseudomonas aeruginosa* [18]. WH is a deciduous shrub or small tree that is native to Northeast USA and Canada. It has been used traditionally by Native Americans to treat skin inflammation, colds and fevers and for healing superficial wounds by applying or ingesting decoctions of the shrub [19,20]. The plant is rich in phenolic compounds, including gallic acid, gallocatechin and epigallocatechin [21]. It also contains a relative abundance of the low molecular weight phenolic component hamamelitannin (HAMA; 2′,5-di-O-galloyl-d-hamamelose), which has been reported to affect staphylococcal biofilm formation by inhibiting both quorum-sensing [22] and toxin production [23]. An earlier study found that HAMA could prevent MRSA infections in vivo [24], thereby indicating suppressive antibacterial effects of the compound on resistant staphylococci. However, other studies of WH-derived compounds or extracts on MRSA or other types of resistant bacteria are lacking, and further studies are required. The aim of this present study was to test WH extracts against a selection of Gram-positive and Gram-negative bacterial species and their antibiotic-resistant counterparts. Included in this study were *S. aureus* and MRSA, *E. coli* and ESBL *E. coli*, and *K. pneumoniae* and ESBL *K. pneumonia*. These bacteria are responsible for many of the deaths caused by bacterial infections, and these species often possess high levels of antibiotic resistance. Gas chromatography–mass spectrometry (GC-MS) analysis of the WH extracts was used to highlight noteworthy compounds in the extracts.

## 2. Results

### 2.1. Antibacterial Activities

**Figure 1:**
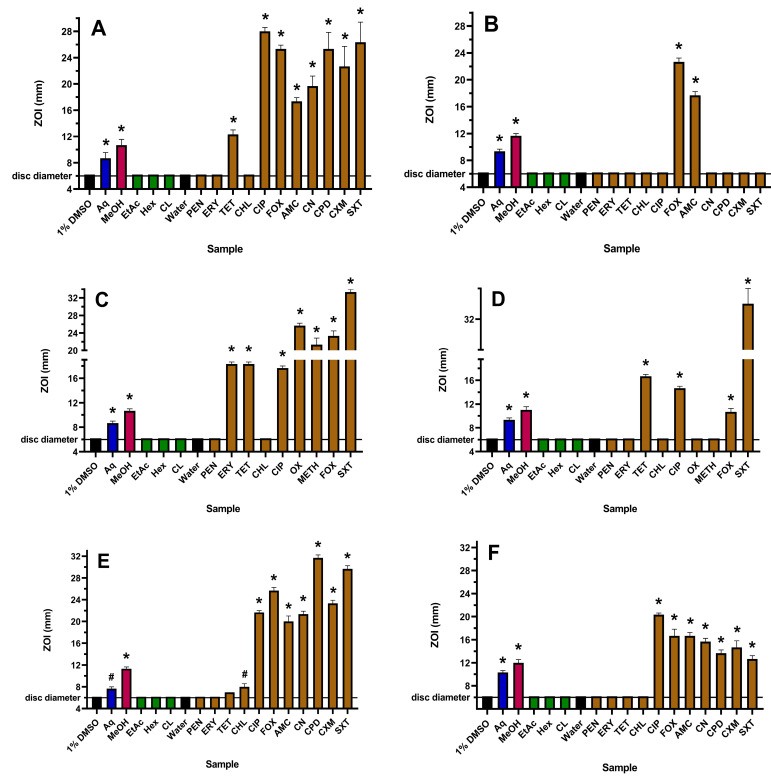
Antibacterial activity of WH extracts and antibiotics on agar against (**A**) *E. coli*, (**B**) ESBL *E. coli*, (**C**) *S. aureus*, (**D**) MRSA, (**E**) *K. pneumoniae* and (**F**) ESBL *K. pneumoniae*, measured as ZOI (mm). For the extract samples (10 µL per disc), Aq = aqueous; MeOH = methanolic; EtAc = ethyl acetate; Hex = hexane; CL = chloroform. Antibiotics tested at 1 µg per disc are PEN = penicillin; ERY = erythromycin; TET = tetracycline; CHL = chloramphenicol; and CIP = ciprofloxacin. OX (oxacillin) and METH (methicillin) were tested at 5 µg per disc, gentamicin (CN) and cefpodoxime (CPD) at 10 µg per disc, AMC (Augmentin) was tested at 15 µg per disc, SXT (trimethoprim/sulfamethoxazole) at 25 µg per disc and FOX (cefoxitin) and cefuroxime (CXM) at 30 µg per disc. The stock concentrations of the WH extracts were 19.7 mg/mL (Aq), 26.8 mg/mL (MeOH), 5.1 mg/mL (EtAc), 8.2 mg/mL (Hex) and 16.4 mg/mL (CL). Results are expressed as mean zones of inhibition of triplicate assays ± SEM. Asterisks indicate results that are significantly different to the relevant negative control (*p* < 0.01); # = not significant.

**Figure 2:**
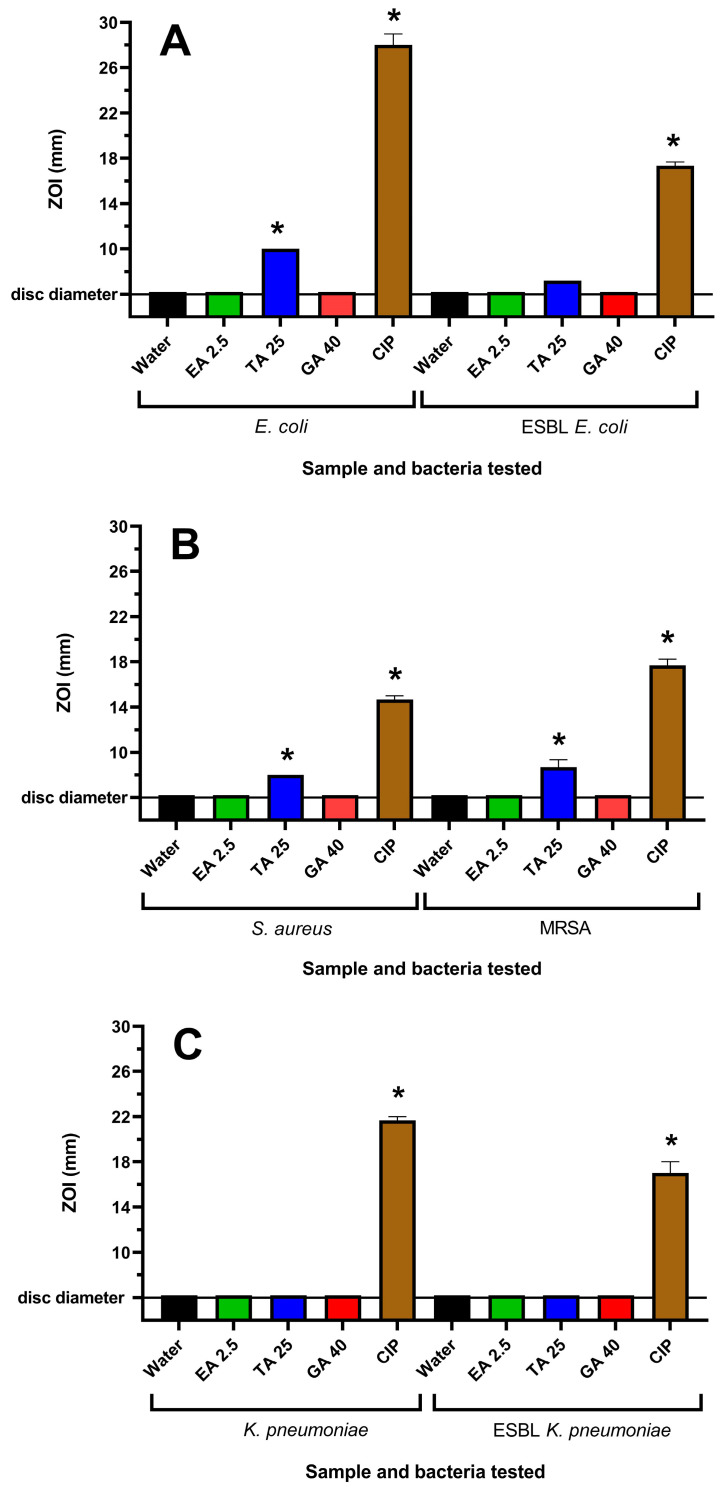
Effects of tannin compounds on (**A**) *E. coli* and ESBL *E. coli*, (**B**) *S. aureus* and MRSA and (**C**) *K. pneumoniae* and ESBL *K. pneumoniae* growth on agar. Ciprofloxacin (1 µg per disc) was used as a positive control, whilst the negative control was water. EA 2.5 = ellagic acid at 2.5 µg per disc; TA 25 = tannic acid at 25 µg per disc; and G 40 = gallic acid at 40 µg per disc. Results are expressed as mean zones of inhibition of triplicate assays ± SEM, and asterisks denote results that are significantly different to the control (*p* < 0.05).

The susceptibility or resistance of all bacterial species and strains used in this study were verified using multiple antibiotics from several classes. Activities on agar, as determined via zones of inhibition (ZOIs) on plates, and in broth, as determined via minimum inhibitory concentration (MIC) values, were generally concordant for each antibiotic (Figure 1, Figure 2 and Table 1). 

The yields following plant extraction with different solvents followed by drying and then resuspension in 1% DMSO were 19.7, 26.8, 5.1, 8.2 and 16.4 mg/mL for the aqueous, methanolic, ethyl acetate, hexane and chloroform extracts, respectively. The WH aqueous and methanolic extracts showed activity towards all six strains used in this study, and in each case, the methanolic extract was more potent than the water extract on both agar (Figure 1) and in liquid broth (Table 1). The ethyl acetate, hexane and chloroform extracts were inactive against all strains using both assay methodologies and were therefore not tested further. Although it was difficult to ascertain differences in the levels of inhibition on agar for the resistant bacteria, the MIC values obtained using microdilution broth assays indicated that the active WH extracts were more potent towards the ESBL *E. coli* compared to sensitive *E. coli* and were more potent towards MRSA compared to *S. aureus*. This interesting finding suggests that these extracts may be more effective in treating infections caused by the resistant *E. coli* and *S. aureus* strains. However, this effect was not observed for ESBL *K. pneumoniae*, where the MIC values for this strain and the sensitive *K. pneumoniae* were similar.

WH extracts have previously been reported to be rich in tannin compounds [25]. Therefore, it was believed that tannins may contribute to the antibacterial activity of the WH extracts. Three tannins, ellagic acid (EA), tannic acid (TA) and gallic acid (GA), were also tested for their antibacterial activities. EA and GA were inactive on agar at all concentrations tested. TA was inactive against *K. pneumoniae* and ESBL *K. pneumoniae*, although it inhibited the growth of *E. coli*, ESBL *E. coli*, *S. aureus* and MRSA (Figure 2). This corresponded to the growth inhibition noted in the broth assays, where noteworthy MIC values of 156–625 µg/mL were obtained against these bacterial strains for TA. However, EA and GA did not inhibit the growth of any of the bacterial strains at the highest concentrations examined.

### 2.2. Fractional Inhibitory Concentration (FIC) Determinations

For each bacterial species tested, combination experiments were conducted if both the plant extract and the antibiotic involved were both inhibitors of growth (and yielded MIC values <4000 µg/mL for the extract or <2.5 μg/mL for the conventional antibiotics) when used alone. FIC values were obtained using 1:1 ratios of the active antibiotics and extracts (Table 2), from which the ΣFIC values were determined. Table 2 shows these values for the combination experiments that were performed. Among these, three additive interactions were observed. These included combinations containing tetracycline and the aqueous WH extract against MRSA, tetracycline and the methanolic WH extract against MRSA, chloramphenicol and the methanolic WH extract against *E. coli*, and ciprofloxacin and the methanolic WH extract against *S. aureus*. All other combinations tested were found to be non-interactive. No antagonistic or synergistic interactions were observed between the WH extracts and antibiotics. 

### 2.3. Qualitative GC-MS Profiling of Extracts

GC-MS was performed on each extract to help correlate activity with the presence of specific phytochemicals (Table 3). A variety of different aliphatic or aromatic compounds were detected. Phthalane was detected in the aqueous and methanolic extracts, whilst several terpenoids (menthol, epoxy-cumene and camphene) were identified in low relative abundances within several extracts. Notably, the dioxolane compounds 2-heptyl-1,3-dioxolane and 1,3-dioxolane-2-methanol were detected in relative abundance in the bioactive WH extracts. Whilst these compounds were detected in all extracts, the overall crude extract yields of the aqueous and methanolic extracts were substantially higher than the relative abundances in the ethyl acetate, chloroform and hexane extracts. It is possible that these compounds, as well as the phthalane that is present in these extracts, may contribute to the antibacterial activities observed in our study. 

## 3. Discussion

The growth of all six of the bacterial species included in this study was inhibited by the aqueous and methanolic WH extracts when tested both on agar and in broth. Moreover, the methanolic extracts were more potent inhibitors of bacterial growth against all species/strains. Contrastingly, the ethyl acetate, hexane and chloroform extracts were inactive. It is likely that these findings may correspond to the variable yields and types of phytochemicals extracted from WH with the various solvents. Generally, lower polarity solvents extract fewer phytochemicals from plant material than polar solvents [26], thereby generating decreased extract yields, as well as a smaller array of phytochemicals. Our previous work has shown that water extracts substantial amounts of a variety of tannins, flavonoids, and other polyphenolics, whilst methanol also extracts many of the same compounds, in addition to other lower polarity phytochemicals [17]. In contrast, the lower polarity solvents ethyl acetate, hexane and chloroform extract fewer phytochemicals and are in low abundance. Taken together, these findings may reflect the relative levels of bacterial growth inhibition in the disc diffusion and MIC assays.

Notably, the WH extracts inhibited the growth of antibiotic-resistant bacterial species with similar potencies to those observed for antibiotic-susceptible bacteria. This indicates that the active WH extract compounds are relatively unaffected by the resistance mechanisms present in MRSA and ESBL bacteria. Thus, the presence of β-lactamase enzymes or mutated peptidoglycan synthesis enzymes does not compromise WH extract potency, suggesting that the mechanisms of action of the compound(s) in the extracts are dissimilar to those of β-lactam drugs. Alternatively, other components in these extracts block the antibiotic-resistance mechanisms in those strains. It also indicates that these WH extracts may possess novel, uncharacterised antibacterial mechanisms that contribute to their activities and are effective despite the presence of β-lactam resistance. This is promising as the antibiotic-resistant bacteria included in this study also have much lower susceptibilities against many different antibiotics of different classes, including β-lactams, macrolides, sulfonamides, fluoroquinolones, aminoglycosides and tetracyclines compared to their susceptible counterparts. Furthermore, the WH extracts were effective against both Gram-positive and Gram-negative bacteria, further highlighting their potential broad-spectrum antibiotic applications.

There are many reports that demonstrate the potential of combinations of plant extracts with conventional antibiotics to enhance the antibacterial activities of the antibiotic components [16]. This was examined in our study by combining the active extracts with conventional antibiotics that were also inhibitory against the bacterial strains tested herein. Most of these combinations yielded indifferent interactions and thus failed to enhance antibacterial activities against the six strains tested. Whilst these combinations provide no benefit over either therapy used independently, they do indicate that the combinations do not decrease the activity of the components and, therefore, are not detrimental to use in combination. This is important, as many users of herbal medicines often use those therapies concurrently with allopathic therapies, often without advising their medical practitioner [27]. Interestingly, additive effects were observed for three combinations (aqueous or methanolic extracts with tetracycline against MRSA; methanolic extracts combined with chloramphenicol against *E. coli*). This indicates that these combinations may be beneficial to enhance the antibacterial efficacy of the therapy against some bacterial strains. Additionally, WH extracts have been found to be non-toxic in brine shrimp toxicity assays [17], further supporting their safe use. Notably, synergistic combinational effects were not detected in this study, although a wider panel of different antibiotics or bacterial species may reveal synergy. Interestingly, an iodine-based teat dip combined with a concentrated commercial witch hazel preparation has been found to synergistically inhibit the growth of *E. coli* and *P. aeruginosa* growth [28], which may be useful in the treatment of mastitis in dairy cattle. WH extracts can also be combined with green tea extracts to synergistically inhibit *S. aureus* growth using a similar assay model [23]. In both cases, the underlying synergy mechanisms remain to be determined. However, we were unable to find reports of synergistic antibacterial activity between WH extracts and conventional antibiotics.

**Figure 3:**
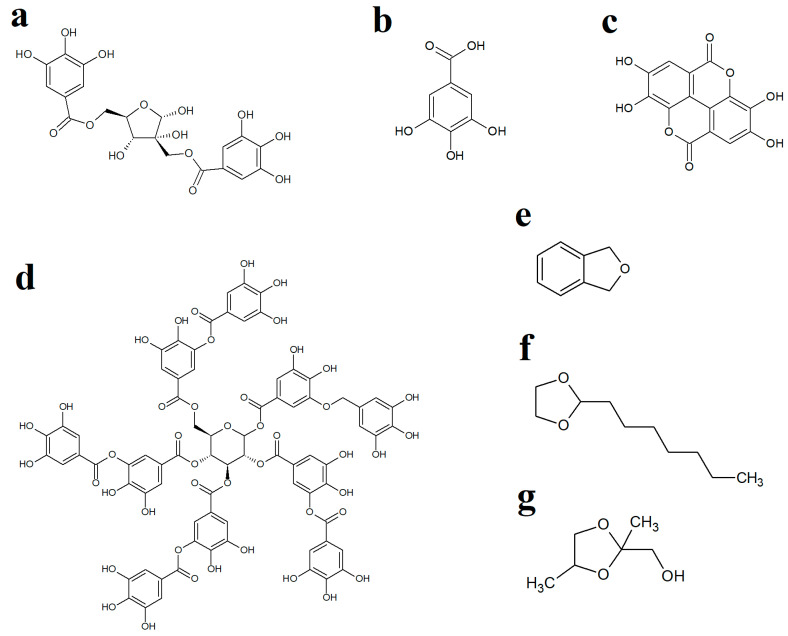
Chemical structures of (**a**) hamamelitannin, (**b**) gallic acid, (**c**) ellagic acid, (**d**) tannic acid, (**e**) phthalane, (**f**) 2-heptyl-1,3-dioxolane and (**g**) 1,3-dioxolane-2-methanol.

WH leaves are a rich source of hydrolysable and condensed tannins [29] and have been reported to contain 3–10% (*w*/*w*) tannin content [30]. Interestingly, one of the main tannins in WH leaves, hamamelitannin (HAMA) (Figure 3a), has been reported to be an ineffective growth inhibitor of several bacteria in vitro [23,31]. WH leaves also contain substantial levels of gallic acid ([21]; Figure 3b) as well as several other tannins, including ellagic acid (Figure 3c) and tannic acid (Figure 3d), which have previously been reported to inhibit the growth of multiple bacteria [32,33]. Therefore, we screened pure gallic, ellagic and tannic acids against the bacterial strains examined herein to determine if these molecules, when used in isolation, could inhibit the growth of bacteria. Interestingly, activity was observed for tannic acid against *E. coli*, ESBL *E. coli*, *S. aureus* and MRSA but not against *K. pneumoniae* and ESBL *K. pneumoniae*. Tannic acid is a complex hydrolysable tannin (Figure 3d), which consists of ten gallic acid moieties esterified (either directly or indirectly) to a central glucose residue. This molecule readily hydrolyses in aqueous solutions (particularly in mildly acidic pHs) to release up to ten gallic acid molecules per tannic acid molecule [34], which may account for the relatively good antibacterial activity of tannic acid. In contrast, free (non-complexed) gallic acid and ellagic acid were inactive at the highest concentrations tested. However, it is noteworthy that ellagic acid has limited solubility in aqueous solutions and was, therefore, only tested at relatively low concentrations (≤625 µg/mL), which may have limited its effects in these assays. However, despite the activity of tannic acid (and the potential activity of higher concentrations of gallic and ellagic acids), it is likely that other extract components also contribute to the activity of the extracts. These findings highlighted the potential for potentiating interactions between extract components and prompted us to identify other extract components using GC/MS. Whilst previous studies have used LC-MS analysis to identify the high- to mid-polarity compounds (including the tannins) [28,29], the mid- to lower-polarity components of WH remain relatively neglected, despite multiple hydrocarbons and terpenoids having noteworthy antibacterial activity [35]. Therefore, we selected GC-MS headspace analysis to identify lower polarity volatile compounds in the extracts. 

Whilst numerous aliphatic and aromatic compounds were detected in the WH extracts, several compounds of particular interest were identified. Phthalane (Figure 3e), also known as isocoumarin, was present in the aqueous and methanolic extracts. The isocoumarins are naturally occurring secondary metabolites in higher plants, as well as in some microbes [36,37]. These compounds have been shown to exert good antibacterial activity towards *S. aureus*, MRSA, *E. coli*, *S. epidermidis*, *H. pylori*, *P. aeruginosa* and *B. subtilis* [38,39,40,41,42,43] and may, therefore, contribute to the antibacterial activity noted for the WH extracts in our study. Postulated mechanisms of isocoumarin action include penicillin-binding protein inhibition in *H. pylori* [42], as well as cell membrane interaction in *S. aureus*, *S. epidermidis* and *E. faecalis* due to their lipophilic nature and planar structure, which assist in bacterial cell wall penetration [44]. However, the specific isocoumarin compound tested in that study (paepalantine) has not been reported to be present in WH leaves. It remains to be determined whether the phthalane detected in our study has similar activities/potencies, and future studies are planned to test this. Other studies have also reported that DNA gyrase of the parasite *Plasmodium falciparum* is inhibited by isocoumarin [45]. However, this may not be the case for the bacteria tested in our study as we did not see enhancement of bacterial growth inhibition by the DNA gyrase inhibitor ciprofloxacin, which would be expected for this class of antibiotics.

Interestingly, another isocoumarin (amicoumarin A) inhibits MRSA growth [46] by interacting with the 16S rRNA within the bacterial 30S ribosomal subunit [47], thereby inhibiting translation. In this present study, two antibiotics (erythromycin and tetracycline) that bind to the bacterial ribosome to inhibit protein synthesis were included. Erythromycin interacts with the 23S rRNA in the 50S ribosomal subunit. It is, therefore, unlikely that isocoumarins (such as amicoumarin A) would affect erythromycin activity, thereby explaining why there was no interaction between WH extracts and this antibiotic in our study. However, tetracycline binds to the 16S portion of the 30S ribosomal subunit, and we did observe potentiation of WH antibacterial activity against MRSA. This intriguing finding warrants further investigation.

Two dioxolane compounds, 2-heptyl-1,3-dioxolane and 1,3-dioxolane-2-methanol, were also detected in the WH extracts. Notably, 1,3-dioxolane derivatives serve as a scaffold for several pharmacologically active compounds, including the antifungal molecules ketoconazole and itraconazole, as well as several other biologically active natural products [48]. Various substituted 1,3-dioxolanes inhibit *E. coli* and *Staphylococcus* growth on nutrient agar [49]. Microdilution broth assays revealed MIC values of different chiral and racemic 1,3-dioxolanes ranging from 156 to 1250 µg/mL against *S. aureus*, *S. epidermidis*, *E. faecalis*, *P. aeruginosa*, as well as against the fungal species *C. albicans* [50]. In contrast, the substituted 1,3-dioxolanes were ineffective inhibitors of *E. coli*, *K. pneumoniae* and *P. mirabilis* growth. The mechanisms of action of the 1,3-dioxolane compounds have not been investigated to date.

It is possible that, while the phthalane and 1,3-dioxolane compounds found in WH may possess antibacterial activities by themselves, they may instead (or in addition) act as potentiators of the activities of other phytochemicals present in WH and of some conventional antibiotics, although this remains to be verified. Future studies should investigate these molecules as new scaffolds for novel antibiotics and/or potentiators of existing antibiotics. The active WH extracts in this present study were effective against both the antibiotic-susceptible and -resistant bacteria. Thus, newly developed drugs (or drug formulations) should ideally possess this property. As part of this approach, phthalane and 1,3-dioxolane compounds should be further characterised and then tested for their inherent antibacterial activities and then examined in combination with other WH phytoconstituents (such as the various tannins, polyphenols, cardiac glycosides, etc.) to determine the antibacterial potencies and the bacteriostatic or bacteriocidal mechanisms by which the WH compounds operate. Additionally, several compounds detected in the GC-MS metabolomics profiling analysis presented in this study were unable to be identified. It is possible that those compounds may also contribute to the antibacterial activity of the extracts examined in this study, either as the antibacterial component or as a potentiator. Further studies are required to identify these compounds and examine their potential activities.

## 4. Materials and Methods

### 4.1. Plant Sources and Extractions

WH leaf fragments (~5 mm in length) were sourced from the US and purchased from Noodles Emporium (Australia). The dried leaf material was stored as voucher specimens (GU2018WHa) at the School of Environment and Science, Griffith University, Australia. One-gram quantities of the plant material were added to individual 50 mL tubes, and 50 mL of either sterile deionised water, methanol, ethyl acetate, hexane or chloroform were added. All organic solvents were obtained from Ajax Fine Chemicals, Australia and were AR grade. Solvent extraction was conducted at room temperature with continuous mixing for 24 h. The extracts were then filtered through Whatman No. 54 filter paper to remove particulate matter. Organic solvents were evaporated by air drying at 45 °C for 48 h, whilst aqueous extracts were freeze dried via lyophilisation at −80 °C in a VirTis sentry 2.0 Bench Top Lyophilizer (SP Scientific, Gardiner NY, USA) for up to 72 h. Extraction yields were determined by weighing the dried extracts, and these were then resuspended in 10 mL of sterile deionised water (containing 1% DMSO). After mild sonication of each suspension (three 20 s pulses at 1 kHz, with 30 s rest between pulses), the extracts were passed through 0.22 mm Millex-GS mixed cellulose ester membrane syringe filter units (Merck Pty. Ltd., Baywater, Australia) and stored at 4 °C in tightly capped polypropylene tubes until required.

### 4.2. Bacterial Cultures

Reference strains for *E. coli* (ATCC 25922), *S. aureus* (ATCC 25923), MRSA (ATCC 43300), *K. pneumoniae* (ATCC 13883) and ESBL *K. pneumoniae* (ATCC 700603) were purchased from the American Type Culture Collection (ATCC, Manassas, VA, USA) and were used in this study. The ESBL-resistant *E. coli* screened herein was a clinical isolate acquired from the Gold Coast University Hospital, Australia. Antibacterial testing conditions conformed to CLSI standardised methods [51]. Powdered dehydrated media was purchased from Oxoid Ltd. (Scoresby, Australia), and the bacterial cultures were maintained at 37 °C in aerobic conditions on Mueller–Hinton (MH) agar for disc diffusion assays, and in MH broth for liquid cultures, except for MRSA, which was grown at 35 °C. For *S. aureus* and MRSA growth, media was supplemented with 2% NaCl. 

### 4.3. Conventional Antibiotics

Penicillin g (potency of 1440–1680 μg/mg), chloramphenicol (≥98% purity by HPLC), erythromycin (potency of ≥850 μg/mg), ciprofloxacin (≥98% purity by HPLC) and tetracycline (≥95% purity by HPLC) were purchased from Sigma-Aldrich (Castle Hill, NSW, Australia) and were used as controls for the disc diffusion and microplate broth microdilution assays. Preloaded discs (Oxoid Ltd., Thebarton, SA, Australia) containing additional antibiotics were used in disc diffusion studies as positive controls to further test the bacteria’s antibiotic susceptibilities on agar. These included oxacillin and methicillin (5 µg per disc), gentamicin and cefpodoxime (10 µg per disc), Augmentin™ (15 µg per disc), trimethoprim/sulfamethoxazole (25 µg per disc at 1:19 ratio), and cefoxitin and cefuroxime (30 µg per disc).

### 4.4. Tannins

The effects of tannins on bacterial growth were also studied. Ellagic acid (EA), tannic acid (TA), and gallic acid (GA) were purchased from Sigma Aldrich Ltd. (Castle Hill, NSW, Australia), and stock solutions prepared in deionised water were used for assays on agar and in broth. Ciprofloxacin (1 µg per disc) was used as the positive control reference antibiotic for the disc diffusion assay. 

### 4.5. Bacterial Growth Inhibition on Agar

Antibiotic susceptibilities of each bacterial species to WH extracts, tannins and conventional antibiotics were initially assessed by modified disc diffusion assays [52,53]. Following incubation for 16–18 h at 37 °C (or 35 °C for MRSA) on agar, the zone of inhibition (ZOI) was measured for each sample (extract or antibiotic) to the nearest whole millimetre. 

### 4.6. Microplate Liquid Dilution MIC Assay

A standard liquid dilution MIC assay using 96-well microtitre plates was used to more precisely quantify the bacterial growth inhibitory activity of the extracts, tannins and conventional antibiotics, and of the extract/antibiotic combinations [53,54]. Following incubation of the microtitre plates overnight at the required temperature for 18–20 h, 40 μL of iodonitrotetrazolium violet (INT) (Sigma-Aldrich Ltd., Castle Hill, NSW, Australia) was added to each well and incubated for a further 4–6 h to allow for full-colour development. The presence of a rusty red colour indicated bacterial growth, whilst yellow wells indicated a lack of growth. MIC values were visually determined as the lowest concentration of extract or antibiotic at which colour development was inhibited. Extract MIC values > 5000 μg/mL were considered inactive; MIC values between 2000 and 5000 μg/mL were considered as low activity; 1000–2000 μg/mL were considered as moderate activity; 400–1000 μg/mL were considered as noteworthy activity; 100–400 μg/mL were considered as good activity; and <100 μg/mL were considered to be high activity [55,56,57,58,59].

### 4.7. Fractional Inhibitory (FIC) and ΣFIC Assessment

Fractional inhibitory concentrations (FIC) could only be determined for combinations where both the extracts and the antibiotics inhibited bacterial growth, with the exception of antibiotics that inhibited growth at the highest concentration of antibiotic tested, since MIC values cannot be measured in these cases. For all other combinations of extracts and antibiotics, a ratio of 50:50 of extract/antibiotic was tested, and interactions between the two components were examined by measuring the sum of fractional inhibitory concentrations (ΣFIC) for each combination. FIC values for each component (A and E) were calculated using the following equations, where A and E represent the antibiotic and extract components, respectively:

FIC (A) = MIC (A in combination with E)/MIC (A independently)

FIC (E) = MIC (E in combination with A)/MIC (E independently)

The ΣFIC was then calculated using the formula ΣFIC = FIC(A) + FIC(E), and the resultant values were classified as synergistic (ΣFIC ≤ 0.5), additive (ΣFIC > 0.5–≤1.0), indifferent (ΣFIC > 1.0–≤4.0) or antagonistic (ΣFIC > 4.0) [60].

### 4.8. GC-MS Profiling Analysis 

The separation, identification and quantification of the relative abundance of individual extract components were performed using a Shimadzu GC-2010 plus (Canby, ON, USA) chromatography system linked to a Shimadzu MS TQ8040 mass selective detector (Canby, ON, USA) mass selective detector system equipped with a Shimadzu AOC-5000 Plus auto-sampler (Canby, ON, USA) using previously optimised parameters [61]. The system used a Supelco (Columbia, MD, USA) solid-phase micro-extraction fibre (SPME) divinyl benzene/carbowax/polydimethyl siloxane (DVB/CAR/PDMS) handling system. Briefly, separation of compounds was achieved using a 5% phenyl, 95% dimethylpolysiloxane (30 m × 0.25 mm id × 0.25 um) capillary column (Restek, Bellefonte, PA, USA). Helium (99.99%) was utilised as the carrier gas at a flow rate of 0.79 mL/min and an injector temperature of 230 °C. The mass spectrometer was operated in the electron ionisation mode at 70 eV, and the individual mass signals were recorded in total ion count (TIC) mode, which was acquired for 45 min, utilising a mass range of 45–450 *m*/*z*. The ChemSpider database enabled putative identification of the individual compounds.

### 4.9. Statistical Analysis

Disc diffusion assays for all samples were conducted in triplicate, and one-way analysis of variance analysis (ANOVA) was used to calculate statistical significance between control and treatment groups or between treatment groups, where a *p* value < 0.05 was considered statistically significant. Although statistical analysis for the liquid dilution microplate assays could not be performed, the reliability of MIC values was ensured by repeating the broth microdilution assays twice on separate days, with two replicates per assay (n = 4), to confirm that the results were reproducible for all extracts, antibiotics and combinations tested.

## 5. Conclusions

New therapies for the treatment of bacterial infections are urgently required. Given the acceleration in the evolution of highly drug-resistant species, a greater focus has been placed on natural products as sources of new antibiotic remedies. Our findings show that WH extracts can inhibit the growth of multi-drug resistant bacteria with similar efficacies to susceptible strains. This suggests that components present within the extracts of this plant may possess novel, hitherto uncharacterised mechanisms of antibacterial action, and several of the compounds identified in this study may be, at least in part, responsible for the activities observed. Future studies will be directed at examining these compounds further as potential antibacterials, and/or as potentiators of conventional antibiotic drugs or other phytochemicals.

## Figures and Tables

**Table 1:** MIC values (µg/mL) for WH extracts, and reference antibiotics and tannins against the bacterial strains tested in this study.

Extract, Antibiotic or Tannin	MIC (µg/mL)					
	*E. coli*	ESBL *E. coli*	*S. aureus ^a^*	MRSA	*K. pneumoniae*	ESBL *K. pneumonia*
WH-Aq	3448	1724	493	431	1724	2463
WH-MeOH	1173	670	251	168	1341	1257
WH-EtAc	>10,000 *	>10,000 *	>10,000 *	>10,000 *	>10,000 *	>10,000 *
WH-Hex	>10,000 *	>10,000 *	>10,000 *	>10,000 *	>10,000 *	>10,000 *
WH-CL	>10,000 *	>10,000 *	>10,000 *	>10,000 *	>10,000 *	>10,000 *
PEN	>2.5 *	>2.5 *	>2.5 *	>2.5 *	>2.5 *	>2.5 *
ERY	>2.5 *	>2.5 *	0.313	>2.5 *	>2.5 *	>2.5 *
TET	0.625	>2.5 *	0.156	0.156	0.625	>2.5 *
CHL	2.5	>2.5 *	>2.5 *	>2.5 *	>2.5 *	>2.5 *
CIP	CND	>2.5 *	0.625	0.625	CND	0.625
OX	NT	NT	0.039	>2.5 *	NT	NT
METH	NT	NT	0.313	>2.5 *	NT	NT
EA	>625 *	>625 *	>625 *	>625 *	>625 *	>625 *
GA	>1000 *	>1000 *	>1000 *	>1000 *	>1000 *	>1000 *
TA	313	625	156	156	>625 *	625

WH = witch hazel. Values followed by an asterisk (*) indicate lack of growth inhibition at the highest concentration of extract, antibiotic or tannin tested. The range of concentrations used in the assays was 0.01–10 mg/mL for the plant extracts, 0.01–2.5 µg/mL for the reference antibiotics and 0.5–1000 µg/mL for the tannins (dependent on their solubility limits in the assays). Water (Aq), methanol (MeOH), ethyl acetate (EtAc), hexane (Hex) and chloroform (CL), PEN (penicillin), ERY (erythromycin), TET (tetracycline), CHL (chloramphenicol), CIP (ciprofloxacin), OX (oxacillin) and METH (methicillin), EA (ellagic acid), GA (gallic acid) and TA (tannic acid). CND = could not be determined, as inhibition was observed at the lowest concentration of antibiotic tested. NT = not tested, as OX and METH are measures of resistance in *S. aureus* strains only. *^a^* = data from Cheesman et al., 2021 [17]. The stock concentrations of the WH extracts were 19.7 mg/mL (Aq), 26.8 mg/mL (MeOH), 5.1 mg/mL (EtAc), 8.2 mg/mL (Hex) and 16.4 mg/mL (CL).

**Table 2:** FIC and ∑FIC values (where relevant) for the combinations of the aqueous (left) and methanolic (right) WH extracts in combination with antibiotics against the bacterial species used in this study.

Extract Solvent	Antibiotic	FIC or ∑FIC Values	Bacterial Strain						Extract Solvent	Antibiotic	FIC or ∑FIC Values	Bacterial Strain					
			*E. coli*	*ESBL E. coli*	*S. aureus^a^*	MRSA	*K. pneumoniae*	ESBL *K. pneumoniae*				*E. coli*	*ESBL E. coli*	*S. aureus^a^*	MRSA	*K. pneumoniae*	ESBL *K. pneumoniae*
**Aqueous**	PEN	FIC_EXT_							**Methanol**	PEN	FIC_EXT_						
		FIC_PEN_									FIC_PEN_						
		∑FIC									∑FIC						
	ERY	FIC_EXT_			0.25					ERY	FIC_EXT_			0.50			
		FIC_ERY_			1.00						FIC_ERY_			2.00			
		∑FIC			1.25						∑FIC			2.50			
	TET	FIC_EXT_	0.25		0.13	0.50	1.00			TET	FIC_EXT_	1.00		0.50	0.50	1.00	
		FIC_TET_	1.00		1.00	0.50	1.00				FIC_TET_	1.00		2.00	0.50	0.50	
		∑FIC	1.25		1.13	**1.00**	2.00				∑FIC	2.00		2.50	**1.00**	1.50	
	CHL	FIC_EXT_	1.00				2.00			CHL	FIC_EXT_	0.5				1.00	
		FIC_CHL_	1.00				0.50				FIC_CHL_	0.25				0.50	
		∑FIC	2.00				2.50				∑FIC	**0.75**				1.50	
	CIP	FIC_EXT_			0.25	1.0		2.00		CIP	FIC_EXT_			0.50	1.0		1.96
		FIC_CIP_			2.00	0.50		0.10			FIC_CIP_			0.25	0.25		2.0
		∑FIC			2.25	1.50		2.10			∑FIC			**0.75**	1.25		2.96

Additive ∑FIC values (>0.5–≤1.0) are shown in blue, bold text. Indifferent values are >1.0–≤4.0. Synergistic (≤0.5) and antagonistic (>4.0) interactions were not observed. Darkened areas indicate combinations that were not tested since the antibiotic was either inactive (MIC > 2.5 µg/mL) or it inhibited bacterial growth at the lowest concentration tested. EXT = extract; PEN = penicillin; ERY = erythromycin; TET = tetracycline; CHL = chloramphenicol; and CIP = ciprofloxacin (CIP). *^a^* = data from Cheesman et al., 2021 [17].

**Table 3:** Qualitative GC-MS analysis of witch hazel extracts and elucidation of empirical formulas. Phthalane and the 1,3-dioxolane compounds are highlighted in **blue, bold text**.

Retention Time (Min)	Empirical Formula	Molecular Mass (Da)	Putative Identification	Relative Abundance (% Total Area)				
				M	W	E	C	H
5.23	C_2_H_6_OS	78	Dimethylsulfoxide		96.15	89.53	85.08	
5.825	Could not be determined				0.05			
7.015	Could not be determined			17.88				
7.047	Could not be determined			29.9				
7.265	Could not be determined			4.18				
8.785	Could not be determined				0.28			
8.824	Could not be determined			0.32				
8.92	Could not be determined			0.15				
9.779	C_8_H_18_O	130	Isobutyl ether		0.29		1.05	
10.034	C_9_H_20_O	144	3,5,5-Trimethylhexanol	0.64			0.55	
10.505	C_8_H_18_O	130	2-Ethyl-1-hexanol	2.15	2.01	6.5	5.21	79.94
11.138	C_6_H_14_O_2_	118	3-Methoxy-3-methylbutanol				0.3	
12.074	C_8_H_8_O	120	**Phthalane**	0.07	0.08			
12.737	C_9_H_18_O	142	Nonanal	0.24				
14.129	C_10_H_20_O_2_	172	2-Ethyl-1-hexyl acetate	0.06				
14.271	C_10_H_20_O_2_	172	**2-Heptyl-1,3-dioxolane**	11.85	0.32	0.98	0.42	7.04
14.707	C_9_H_20_O	144	1-Nonanol	0.02				
14.817	C_10_H_20_O	156	Menthol		0.05			
15.635	C_11_H_16_O_2_	164	5,6,7,8,9-octahydro-2H-benzo[a]cyclohepten-2-one				0.13	
15.718	C_10_H_20_O	156	Decanal	0.06			0.13	
15.837	Could not be determined			0.1	0.07	0.48	4.98	5.46
16.041	C_9_H_10_O	134	Epoxy-cumene	0.04		0.77	0.5	
17.155	C_14_H_22_	190	1,3-Di-tert-butylbenzene	0.04		0.08		
17.308	C_10_H_18_O	154	trans-2-Decenal	0.03				
17.93	C_10_H_16_	136	Camphene	0.1				
19.068	Could not be determined					0.08	0.04	1.11
19.144	C_4_H_8_O_3_	104	**1,3-Dioxolane-2-methanol**	1.91	0.08	0.19	0.07	2.22
19.849	C_16_H_30_O_4_	286	2,2,4-Trimethyl-1,3-pentanediol diisobutyrate	0.02	0.08	0.22	0.1	
20.094	C_12_H_22_O	182	(E)-2-Dodecen-1-al	0.05				
20.42	C_12_H_24_O_3_	216	1,3-Pentanediol, 2,2,4-trimethyl-, 1-isobutyrate	0.06	0.16	0.46	0.21	0.58
25.386	Could not be determined			0.16		0.15	0.22	2.23
26.127	C_11_H_16_O_2_	180	2,6,6-Trimethyl-2-hydroxycyclohexylidene) acetic acid lactone	0.04				
26.135	C_11_H_16_O_2_	180	2(4H)-Benzofuranone, 5,6,7,7a-tetrahydro-4,4,7a-trimethyl-		0.05			
28.034	C_16_H_30_O_4_	286	2,2,4-Trimethyl-1,3-pentanediol diisobutyrate	0.1	0.15	0.26	0.17	1.42

The relative abundance is a measure of the area under the peak expressed as a % of the total area under all chromatographic peaks. M = methanolic extract; W = aqueous extract; E = ethyl acetate extract; C = chloroform extract; H = hexane extract.

## Data Availability

Data are either presented within the manuscript or are available from the corresponding author upon reasonable request.

## References

[1] O’Neill J. (2016). Tackling Drug-Resistant Infections Globally: Final Report and Recommendations. The Review on Antimicrobial Resistance. https://amr-review.org/sites/default/files/160518_Final%20paper_with%20cover.pdf.

[2] World Health Organization (2021). Global Antimicrobial Resistance and Use Surveillance System (GLASS) Report 2021.

[3] D’Andrea M.M., Fraziano M., Thaller M.C., Rossolini G.M. (2019). The urgent need for novel antimicrobial agents and strategies to fight antibiotic resistance. Antibiotics.

[4] Bassetti S., Tschudin-Sutter S., Egli A., Osthoff M. (2022). Optimizing antibiotic therapies to reduce the risk of bacterial resistance. Eur. J. Intern. Med..

[5] UNICEF (2019). Levels and Trends in Child Mortality: Report 2019..

[6] Chaurasia S., Sivanandan S., Agarwal R., Ellis S., Sharland M., Sankar M.J. (2019). Neonatal sepsis in South Asia: Huge burden and spiralling antimicrobial resistance. BMJ.

[7] Álvarez A., Fernández L., Gutiérrez D., Iglesias B., Rodríguez A., García P. (2019). Methicillin-resistant *Staphylococcus aureus* in hospitals: Latest trends and treatments based on bacteriophages. J. Clin. Microbiol..

[8] Romagnoli A., Papucci S.L., Aletti A., Chiossone A., Pigozzi F., Sguassero Y. (2021). Community-acquired methicillin-resistant *Staphylococcus aureus* pneumonia in a children’s hospital. Our ten-year experience. Arch. Argent. Pediatr..

[9] Ali M.F., Marzouq M.A., Hussein S.A., Salman B.I. (2021). A bio-analytically validated HPLC-UV method for simultaneous determination of doripenem and ertapenem in pharmaceutical dosage forms and human plasma: A dual carbapenem regimen for treatment of drug-resistant strain of *Klebsiella pneumoniae*. RSC Adv..

[10] Levine M.M., Nasrin D., Acácio S., Bassat Q., Powell H., Tennant S.M., Sow S.O., Sur D., Zaidi A.K., Faruque A.S. (2020). Diarrhoeal disease and subsequent risk of death in infants and children residing in low-income and middle-income countries: Analysis of the GEMS case-control study and 12-month GEMS-1A follow-on study. Lancet Glob. Health..

[11] Yigit H., Queenan A.M., Anderson G.J., Domenech-Sanchez A., Biddle J.W., Steward C.D., Alberti S., Bush K., Tenover F.C. (2001). Novel carbapenem-hydrolyzing β-lactamase, KPC-1, from a carbapenem-resistant strain of *Klebsiella pneumoniae*. Antimicrob. Agents Chemother..

[12] McDanel J., Schweizer M., Crabb V., Nelson R., Samore M., Khader K., Blevins A.E., Diekema D., Chiang H.Y., Nair R. (2017). Incidence of extended-spectrum β-lactamase (ESBL)-producing *Escherichia coli* and *Klebsiella* infections in the United States: A systematic literature review. Infect. Control Hosp. Epidemiol..

[13] Kawamura K., Nagano N., Suzuki M., Wachino J.I., Kimura K., Arakawa Y. (2017). ESBL-producing *Escherichia coli* and its rapid rise among healthy people. Food Saf..

[14] Lam M.M., Wyres K.L., Wick R.R., Judd L.M., Fostervold A., Holt K.E., Löhr I.H. (2019). Convergence of virulence and MDR in a single plasmid vector in MDR *Klebsiella pneumoniae* ST15. J. Antimicrob. Chemother..

[15] Ding Y., Wang Y., Hsia Y., Sharland M., Heath P.T. (2019). Systematic review of carbapenem-resistant Enterobacteriaceae causing neonatal sepsis in China. Ann. Clin. Microbiol. Antimicrob..

[16] Cheesman M.J., Ilanko A., Blonk B., Cock I.E. (2017). Developing new antimicrobial therapies: Are synergistic combinations of plant extracts/compounds with conventional antibiotics the solution?. Pharmacogn. Rev..

[17] Cheesman M.J., Alcorn S., Verma V., Cock I.E. (2021). An assessment of the growth inhibition profiles of *Hamamelis virginiana* L. extracts against *Streptococcus* and *Staphylococcus* spp. J. Tradit. Complement. Med..

[18] Cheesman M.J., Alcorn S.R., Cock I.E. (2023). Effects of *Hamamelis virginiana* L. extracts on *Pseudomonas aeruginosa* growth and antagonism of ciprofloxacin. Pharmacogn. Commun..

[19] Korting H.C., Schäfer-Korting M., Hart H., Laux P., Schmid M. (1993). Anti-inflammatory activity of hamamelis distillate applied topically to the skin. Eur. J. Clin. Pharmacol..

[20] Moerman D.E. (1986). Medicinal Plants of Native America, Part 1.

[21] Wang H., Provan G.J., Helliwell K. (2003). Determination of hamamelitannin, catechins and gallic acid in witch hazel bark, twig and leaf by HPLC. J. Pharm. Biomed. Anal..

[22] Brackman G., Breyne K., De Rycke R., Vermote A., Van Nieuwerburgh F., Meyer E., Van Calenbergh S., Coenye T. (2016). The quorum sensing inhibitor hamamelitannin increases antibiotic susceptibility of *Staphylococcus aureus* biofilms by affecting peptidoglycan biosynthesis and eDNA release. Sci. Rep..

[23] Rasooly R., Molnar A., Choi H.Y., Do P., Racicot K., Apostolidis E. (2019). In-vitro inhibition of staphylococcal pathogenesis by witch-hazel and green tea extracts. Antibiotics.

[24] Kiran M.D., Adikesavan N.V., Cirioni O., Giacometti A., Silvestri C., Scalise G., Ghiselli R., Saba V., Orlando F., Shoham M. (2008). Discovery of a quorum-sensing inhibitor of drug-resistant staphylococcal infections by structure-based virtual screening. Mol. Pharmacol..

[25] Abbas T.F., Abbas M.F., Lafta A.J. (2020). Antibacterial activity and medical properties of Witch Hazel *Hamamelis virginiana*. Ann. Trop. Med. Public Health.

[26] Eloff J.N. (1998). Which extractant should be used for the screening and isolation of antimicrobial components from plants?. J. Ethnopharmacol..

[27] Ullah K., Parekh A.D., Shaikh O.A., Khan M., Ochani S. (2023). Acute liver failure secondary to the use of unmonitored drugs and herbal supplements: An underreported and serious issue. Ir. J. Med. Sci..

[28] Rasooly R., Molnar A., Do P., Morroni G., Brescini L., Cirioni O., Giacometti A., Apostolidis E. (2020). Witch hazel significantly improves the efficacy of commercially available teat dips. Pathogens.

[29] Vennat B., Pourrat H., Pouget M.P., Gross D., Pourrat A. (1988). Tannins from *Hamamelis virginiana*: Identification of proanthocyanidins and hamamelitannin quantification in leaf, bark, and stem extracts. Planta Med..

[30] Piazza S., Martinelli G., Vrhovsek U., Masuero D., Fumagalli M., Magnavacca A., Pozzoli C., Canilli L., Terno M., Angarano M. (2022). Anti-inflammatory and anti-acne effects of *Hamamelis virginiana* bark in human keratinocytes. Antioxidants.

[31] Leong C., Schmid B., Buttafuoco A., Glatz M., Bosshard P.P. (2019). In vitro efficacy of antifungal agents alone and in shampoo formulation against dandruff-associated *Malassezia* spp. and *Staphylococcus* spp. Int. J. Cosmet. Sci..

[32] Akiyama H., Fujii K., Yamasaki O., Oono T., Iwatsuki K. (2001). Antibacterial action of several tannins against *Staphylococcus aureus*. J. Antimicrob. Chemother..

[33] Farha A.K., Yang Q.Q., Kim G., Li H.B., Zhu F., Liu H.Y., Gan R.Y., Corke H. (2020). Tannins as an alternative to antibiotics. Food Biosci..

[34] Lu L.L., Lu X.Y., Ma N. (2008). Kinetics of non-catalyzed hydrolysis of tannin in high temperature liquid water. J. Zhejiang Univ. Sci. B..

[35] Sharma A., Biharee A., Kumar A., Jaitak V. (2020). Antimicrobial terpenoids as a potential substitute in overcoming antimicrobial resistance. Curr. Drug Targets..

[36] Saeed A. (2016). Isocoumarins, miraculous natural products blessed with diverse pharmacological activities. Eur. J. Med. Chem..

[37] Shabir G., Saeed A., El-Seedi H.R. (2021). Natural isocoumarins: Structural styles and biological activities, the revelations carry on…. Phytochemistry.

[38] Chen S., Liu Y., Liu Z., Cai R., Lu Y., Huang X., She Z. (2016). Isocoumarins and benzofurans from the mangrove endophytic fungus *Talaromyces amestolkiae* possess α-glucosidase inhibitory and antibacterial activities. RSC Adv..

[39] Park H.B., Perez C.E., Perry E.K., Crawford J.M. (2016). Activating and attenuating the amicoumacin antibiotics. Molecules.

[40] Huang G.L., Zhou X.M., Bai M., Liu Y.X., Zhao Y.L., Luo Y.P., Niu Y.Y., Zheng C.J., Chen G.Y. (2016). Dihydroisocoumarins from the mangrove-derived fungus *Penicillium citrinum*. Mar. Drugs.

[41] Lei H., Lin X., Han L., Ma J., Ma Q., Zhong J., Liu Y., Sun T., Wang J., Huang X. (2017). New metabolites and bioactive chlorinated benzophenone derivatives produced by a marine-derived fungus *Pestalotiopsis heterocornis*. Mar. Drugs.

[42] Damasceno J.P.L., Rodrigues R.P., Goncalves R.D.C.R., Kitagawa R.R. (2017). Anti-*Helicobacter pylori* activity of isocoumarin paepalantine: Morphological and molecular docking analysis. Molecules.

[43] Gu B.B., Tang J., Jiao W.H., Li L., Sun F., Wang S.P., Yang F., Lin H.W. (2018). Azaphilone and isocoumarin derivatives from the sponge-derived fungus *Eupenicillium* sp. 6A-9. Tetrahedron Lett..

[44] Devienne K.F., Raddi M.G., Coelho R.G., Vilegas W. (2005). Structure-–antimicrobial activity of some natural isocoumarins and their analogues. Phytomedicine.

[45] Koppula P., Purohit N. (2013). Synthesis of new biologically active triazolo, tetrazolo and coumarinoyl derivatives of isocoumarins. Org. Commun..

[46] Lama A., Pané-Farré J., Chon T., Wiersma A.M., Sit C.S., Vederas J.C., Hecker M., Nakano M.M. (2012). Response of methicillin-resistant *Staphylococcus aureus* to amicoumacin A. PLoS ONE.

[47] Polikanov Y.S., Osterman I.A., Szal T., Tashlitsky V.N., Serebryakova M.V., Kusochek P., Bulkley D., Malanicheva I.A., Efimenko T.A., Efremenkova O.V. (2014). Amicoumacin A inhibits translation by stabilizing mRNA interaction with the ribosome. Mol. Cell.

[48] Gupta R.R., Kumar M., Gupta V. (2013). Heterocyclic Chemistry: Volume II: Five-Membered Heterocycles.

[49] Ovsyannikova M.N., Vol’Eva V.B., Belostotskaya I.S., Komissarova N.L., Malkova A.V., Kurkovskaya L.N. (2013). Antibacterial activity of substituted 1, 3-dioxolanes. Pharm. Chem. J..

[50] Küçük H.B., Yusufoğlu A., Mataracı E., Döşler S. (2011). Synthesis and biological activity of new 1, 3-dioxolanes as potential antibacterial and antifungal compounds. Molecules.

[51] Clinical and Laboratory Standards Institute (2023). Clinical and Laboratory Standards Institute. Clinical and Laboratory Standards Institute CLSI Document M100. Performance Standards for Antimicrobial Susceptibility Testing.

[52] Wright M.H., Shalom J., Matthews B., Greene A.C., Cock I.E. (2019). *Terminalia ferdinandiana* Exell: Extracts inhibit *Shewanella* spp. growth and prevent fish spoilage. Food Microbiol..

[53] Tiwana G., Cock I.E., White A., Cheesman M.J. (2020). Use of specific combinations of the triphala plant component extracts to potentiate the inhibition of gastrointestinal bacterial growth. J. Ethnopharmacol..

[54] Hübsch Z., Van Zyl R.L., Cock I.E., Van Vuuren S.F. (2014). Interactive antimicrobial and toxicity profiles of conventional antimicrobials with Southern African medicinal plants. S. Afr. J. Bot..

[55] Eloff J.N. (1998). A sensitive and quick microplate method to determine the minimal inhibitory concentration of plant extracts for bacteria. Planta Med..

[56] Eloff J.N. (2019). Avoiding pitfalls in determining antimicrobial activity of plant extracts and publishing the results. BMC Complement. Altern. Med..

[57] Eloff J.N. (2004). Quantification the bioactivity of plant extracts during screening and bioassay guided fractionation. Phytomedicine.

[58] Mogana R., Adhikari A., Tzar M.N., Ramliza R., Wiart C. (2020). Antibacterial activities of the extracts, fractions and isolated compounds from *Canarium patentinervium* Miq. against bacterial clinical isolates. BMC Complement. Med. Ther..

[59] Silva A.C., Santana E.F., Saraiva A.M., Coutinho F.N., Castro R.H., Pisciottano M.N., Amorim E.L., Albuquerque U.P. (2013). Which approach is more effective in the selection of plants with antimicrobial activity?. Evid. Based Complementary Altern. Med..

[60] Doern C.D. (2014). When does 2 plus 2 equal 5? A review of antimicrobial synergy testing. J. Clin. Microbiol..

[61] Shalom J., Cock I.E. (2018). *Terminalia ferdinandiana* Exell. fruit and leaf extracts inhibit proliferation and induce apoptosis in selected human cancer cell lines. Nutr. Cancer.

